# Women’s Perceptions of Participation in an Extended Contact Text Message–Based Weight Loss Intervention: An Explorative Study

**DOI:** 10.2196/mhealth.6325

**Published:** 2017-02-27

**Authors:** Jennifer R Job, Lauren C Spark, Brianna S Fjeldsoe, Elizabeth G Eakin, Marina M Reeves

**Affiliations:** ^1^ Cancer Prevention Research Centre School of Public Health The University of Queensland Brisbane Australia

**Keywords:** exercise, diet, text messaging, qualitative research, overweight, obesity, weight loss

## Abstract

**Background:**

Extending contact with participants after the end of an initial weight loss intervention has been shown to lead to maintained weight loss and related behavioral change. Mobile phone text messaging (short message service, SMS) offers a low-cost and efficacious method to deliver extended contact. In this rapidly developing area, formative work is required to understand user perspectives of text message technology. An extended contact intervention delivered by text messages following an initial telephone-delivered weight loss intervention in breast cancer survivors provided this opportunity.

**Objective:**

The aim of this study was to qualitatively explore women’s perceptions of participation in an extended contact intervention using text messaging to support long-term weight loss, physical activity, and dietary behavioral change.

**Methods:**

Following the end of an initial 6-month randomized controlled trial of a telephone-delivered weight loss intervention (versus usual care), participants received a 6-month extended contact intervention via tailored text messages. Participant perceptions of the different types of text messages, the content, tailoring, timing, and frequency of the text messages, and the length of the intervention were assessed through semistructured interviews conducted after the extended contact intervention. The interviews were transcribed verbatim and analyzed with key themes identified.

**Results:**

Participants (n=27) were a mean age of 56.0 years (SD 7.8) and mean body mass index of 30.4 kg/m2 (SD 4.2) and were at a mean of 16.1 months (SD 3.1) postdiagnosis at study baseline. Participants perceived the text messages to be useful behavioral prompts and felt the messages kept them accountable to their behavioral change goals. The individual tailoring of the text message content and schedules was a key to the acceptability of the messages; however, some women preferred the support and real-time discussion via telephone calls (during the initial intervention) compared with the text messages (during the extended contact intervention).

**Conclusions:**

Text message support was perceived as acceptable for the majority of women as a way of extending intervention contact for weight loss and behavioral maintenance. Text messages supported the maintenance of healthy behaviors established in the intervention phase and kept the women accountable to their goals. A combination of telephone calls and text message support was suggested as a more acceptable option for some of the women for an extended contact intervention.

## Introduction

With increasing rates of overweight and obesity worldwide, efforts to promote weight loss and weight loss maintenance have become important aspects of chronic disease prevention and management. Weight loss maintenance is a particular challenge, with weight regains of nearly 50% common in the first year following a weight loss intervention [1]. Research has shown that extending contact beyond an initial weight loss intervention is needed to achieve weight loss maintenance [2-5]. Thus, finding acceptable, efficient, and cost-effective methods to deliver such extended contacts is essential to enable broad population reach [6].

With the development of technology, telephone, text messages (short message service, SMS), email, and Internet have been used to deliver more accessible weight loss interventions [7,8]. These mobile health (mHealth) interventions provide less resource-intensive methods for delivering broad-reach, extended contact for maintaining weight loss.

Text message technology in particular offers a potentially cost-effective method for extending contact [9]. Content and timing of text messages can be tailored to individuals in order to prompt real-time behavior. Participants can give feedback on progress and, in return, receive automated responses.

Recent studies examining the efficacy of text message–delivered, extended contact weight loss maintenance interventions [9-13] are not universally positive. Although the majority of participants find text messaging helpful, with participants generally satisfied with the text messages and the support that they provided [10-14], further intervention development is needed. Qualitative investigation is particularly useful for further intervention refinement, and qualitative data, particularly on extended contact, text message–delivered interventions has been limited to date. Two qualitative investigations have examined the acceptability of extended contact text message interventions following an initial weight loss intervention in an adolescent and a general adult, overweight population [12,14]. Untailored, one-way text messages were used for the study in the adult population [12], whereas in the other, adolescents received semitailored, two-way text messages [14]. The importance of tailoring the content and timing of the text messages to individual needs was a theme that emerged from both studies as an area that could be improved. Both of these extended contact interventions were relatively short (1-3 months) in duration and feedback supported extending the length of extended contact. An in-depth understanding of the acceptability of longer duration (6-month) extended contact, tailored, two-way, text message interventions is warranted.

In a recent study, we examined the feasibility and pre-post efficacy of a 6-month text message–delivered extended contact intervention in promoting the maintenance of weight loss and physical activity and dietary behavioral change in breast cancer survivors who completed an initial 6-month telephone-delivered weight loss intervention [13]. Overall, the results from this pilot study suggested that this highly tailored text message–delivered, extended contact intervention was feasible to deliver and that it might have helped to attenuate weight regain and promote the maintenance of long-term changes in physical activity. Furthermore, 80% (20/25) of women found the intervention helpful (based on a single satisfaction item). Breast cancer survivors are a subgroup of women where weight management, increasing physical activity, and improving dietary quality are encouraged to improve cancer outcomes as well as overall chronic disease risk [15]. This study reported on the results of qualitative interviews in this sample of breast cancer survivors that sought to explore women’s perceptions of the acceptability of text message content, timing and frequency, level of automation, and length of contact. The results will inform further development of extended contact text message interventions.

## Methods

### Study Design

The aims of this study were addressed in the context of the Living Well after Breast Cancer feasibility trial, a randomized controlled pilot study evaluating a 6-month telephone-delivered weight loss intervention (versus usual care) for breast cancer survivors. Women who completed the 6-month telephone-delivered intervention were invited to take part in a 6-month extended contact intervention delivered via text messages. The methods and results of the initial 6-month randomized controlled trial [16] and the pre-post extended contact text message intervention have been previously reported [13].

### Participant Recruitment

Briefly, 90 women diagnosed with stage I-III breast cancer in the previous 9-18 months (age 18-75 years; body mass index 25-40 kg/m^2^) were recruited from a state-based cancer registry. Following baseline assessment, participants were randomized to either a 6-month telephone-delivered intervention (n=45) or usual care group (n=45). Of the 40 women who completed the 6-month intervention and assessment, 37 owned a mobile phone and 30 consented to participate in the extended contact intervention (81% of those eligible). Of the 7 who did not take up the extended contact intervention, 1 participant did not feel she needed the support, 1 used her mobile phone for emergencies only, 3 did not join for health or family reasons, and 2 were not contactable for consent [13]. The qualitative interview was completed by 27 women (90% of those consenting) after completion of the extended contact intervention.

### Intervention

The initial 6-month telephone-delivered intervention included up to 16 calls from an accredited dietitian (coach) and posted printed project materials. The intervention focused on supporting participants to achieve modest weight loss of 5-10% through increasing moderate-to-vigorous physical activity, reducing energy intake (as well as improving dietary quality), and with an emphasis on behavioral change strategies. Participants were also offered text messages at their second call to support and prompt behavioral change in between intervention calls. Over half (57%, 17/30) of the women who participated in the extended contact intervention had received text messages during the initial 6-month intervention.

The extended contact intervention was delivered via individually tailored text messages. The aim was to maintain improvements in physical activity, dietary intake, and body weight that had been established in the initial intervention.

At the start of the extended contact intervention, participants received a telephone call from their coach to gather information to determine individual preferences for the content, timing, and frequency of text messages. A weight loss or weight maintenance goal was set, and two shorter-term goals that focused on either, physical activity and dietary behaviors or both, were established with the coach.

Each participant received a minimum of 21 text messages over the 6-month intervention, including at least twelve to prompt self-monitoring of weight, 3 text messages to reset goals for weight, and 3 text messages to reset goals for each of their physical activity and dietary behavior targets. In addition, participants could choose to receive text messages to check on goal attainment (maximum n=24 for each behavior) and to cue for planned behaviors (maximum n=48 for each behavior). Participants were asked to respond “yes” or “no” to the goal checks. An automated response was then sent in reply. In some cases, participants responded to these goal checks in a way that was not recognized by the system as “yes” or “no.” In such cases, the participant reply was reviewed by a member of the research team and an individual text message was sent to the participant. The lag time for researcher-generated (nonautomated) responses varied considerably (median: 57.5 minutes; minimum-maximum: 2 minutes to 3 days, 9 hours), depending on whether participant goal check replies were received during work hours or after hours. Examples of each type of text message are shown in Table 1.

**Table 1:** Examples of the types of text messages sent during the intervention.

Text message type	Example of text messages
Behavioral prompt	U have already come a long way Karen. Keep it up & walk 3x30mins this week. Amy
Prompt weight self- monitoring	Hi Karen. Have u checked ur weight lately? Make time 2 weigh yourself today. Amy
Goal check	On top of things Karen? Did u eat salad 5x this week? Text me back yes or no & let me know. Amy
Goal check reply to yes	Great work Karen. Be prepared & plan ahead 4 this week so u can achieve ur goal again this week. Put ur plans into action. Amy
Goal check reply to no	Its ok Karen - its natural 2 have a slip every now & again. But remember every day is a new beginning so get back on track - I know u can do it. Amy
Goal reset: weight	If u have a new weight goal 4 the next 6 weeks text it 2 me Karen so I know & can keep supporting u. Amy
Goal reset	Are u focusing on ur portion sizes & making sure u eat breakfast Karen? If u want 2 change ur diet goal text me back & let me know. Amy

After 12 weeks of the extended contact intervention, participants received a telephone call from their coach to check on the relevancy of the text messages and to adjust goals, frequency, and the types of text messages as required.

### Data Collection and Outcomes

Interviews were conducted from March to November 2012 by one investigator (LS) not involved in intervention delivery. A semistructured interview guide was used during the interview, and the focus of the questions surrounded women’s likes and dislikes about the language, content, and timing of the text messages, and their suggestions for improving text message support for physical activity and healthy eating (Multimedia Appendix 1). Interviews were audio-recorded, transcribed verbatim using word processing software, and the transcript accuracy was checked.

### Data Analysis

Thematic analysis was conducted by 2 investigators (JJ) and (LS) and a research assistant independently, with initial themes generated. A fourth investigator (BF) then reviewed the transcripts to ensure appropriate theme formation.

## Results

### Principal Findings and Baseline Characteristics of Participants

In total, 27 interviews were conducted (mean duration: 9 minutes 40 seconds, range: 1:53-19:56). Participants were all white with a mean age of 56.0 years (SD 12.0) and mean BMI 30.4 kg/m^2^ (SD 4.2) and were at a mean of 16.1 months (SD 3.1) postdiagnosis at study baseline. The majority of women were married or in a de facto relationship (85%, 23/27), employed full or part-time (67%, 18/27), with no dependent children (70%, 19/27) and post-menopausal at breast cancer diagnosis (63%, 17/27). All women had undergone breast cancer surgery, and most had also been treated with chemotherapy (63%, 17/27), radiation therapy (78%, 21/27), and endocrine therapy (74%, 20/27). Participants who completed interviews at 12 months were largely representative of the group of intervention participants at baseline (n=45; Table 2).

**Table 2:** Baseline characteristics of all intervention participants (n=45) and those who received extended care and were interviewed for the qualitative analysis (n=27).

Baseline characteristics				All intervention participants (n=45)		Participants interviewed (n=27)		Participants not interviewed (n=18)^a^		*P* ^b^
n				45		27		18		
Age (years), mean (SD)				56.4 (9.0)		56.0 (7.8)		56.9 (10.8)		.76
Body mass index (kg/m^2^), mean (SD)				30.6 (4.3)		30.4 (4.2)		30.9 (4.5)		.66
White, n (%)				43 (96)		27 (100)		16 (89)		.16
Married or de facto, n (%)				35 (78)		23 (85)		12 (67)		.17
Children (nil <18 years at home), n (%)				35 (78)		19 (70)		16 (81)		.27
**Household income, n (%)**										
	≥AU$1578 per week^c^			11 (24)		7 (26)		4 (22)		>.99
	<AU$1578 per week or refused or missing or don’t know			34 (76)		20 (74)		15 (78)		>.99
Completed education beyond high school, n (%)				33 (73)		20 (74)		13 (72)		>.99
Employed (full-time, part-time, casual), n (%)				28 (62)		18 (67)		10 (56)		.54
Postmenopausal at diagnosis, n (%)				24 (53)		17 (63)		7 (39)		.14
Time since diagnosis (months), mean (SD)				16.1 (3.0)		16.1 (3.1)		16.1 (2.9)		.97
Chemotherapy, n (%)				29 (64)		17 (63)		12 (67)		>.99
Radiation, n (%)				35 (78)		21 (78)		14 (78)		>.99
Endocrine therapy, n (%)				32 (71)		20 (74)		12 (67)		.74

^a^ Not interviewed: not part of the extended care sample (n=15), unable to contact or declined qualitative interview (n=3).^b^*P* value for difference in interviewed versus not interviewed by independent-samples *t* test (continuous) or chi-square test (categories).^c^Top 2 quintiles for household income based on the Australian population at the most recent census [17].

Of those who received the text messages and completed the interview, 42% were frequent text message users before the baseline intervention, 12% used texts to “some degree,” and 46% were not text message users.

The 27 women who were interviewed received an average of 7 text messages per fortnight (range 2-11 texts); generally, 1 x weight self-monitoring, 3 x planned behavior prompts, 2 x goal checks, and 1 x tailored goal check reply and 1-2 goal resets each month. There was a 67% response rate to goal checks with participants replying to every 2 in 3 goal check messages and a 20% response rate to goal reset messages.

### Overall Perceptions of the Extended Contact Intervention

Women found the extended contact text message intervention highly acceptable. The majority of women found the number and timing of texts suitable, as it was based on their preferences determined through the initial tailoring interview and updated at the 12-week call. Participants generally reported reading all the text messages, although they might not have been read them straight away, and for some, the reading of the text messages tapered toward the end:

I always read the messages...I did take on what you’re saying in the beginning. You really read the message, but in the end...I suppose I slightly less focused on reading the whole thing, just picking up the gist of it.

6: 47 years

Participants felt 6 months was a good duration for an extended contact intervention as “you’re learning to stand on your own two feet but you’re not totally alone. You know that you’ve got support there….” (12: 49 years) while others felt extending the program longer than 6 months would be beneficial “I would probably go longer” (5: 44 years).

Some commented that they found the texts more convenient than telephone calls and more time efficient. For this group of women, there were rarely issues with the language or abbreviations used in the texts. “I found her abbreviations quite easy to understand” (22: 65 years).

There were no patterns of differences in acceptability between those who had received text messages during the initial intervention and those who did not.

### Text Messages Acted as a Prompt for Clients

A common theme from the interviews was that the text messages were a prompt (or reminder) of topics covered, and particularly of specific skills taught during the initial intervention:

I like the ones that said specifically, ‘have you remembered to weigh yourself this week’…‘Remember to record what you eat,’ those sort of things cause they’re practical things that came straight out of the book, and it brings you back to...a, b, and c

6: 47 years

The text messages also reminded women of the goals they had set:

*I think they were good...To keep one on the straight and narrow. Just a reminder and a good follow up. Yes, to keep me on track* [18: 62 years]

It’s like...the voice in your head...telling you what to do

14: 51 years

### Text Messages Maintained the Perception of Accountability for Clients

The text messages provided a continuation of the “check-in” that the coach had provided over the telephone during the initial intervention. Participants found the goal check text messages kept them accountable to the program aims. “I’d be more aware of what I’m putting in my mouth, because I haven’t reached my goal yet” (14: 51 years).

Responding to text messages about whether weight, diet, or activity goals had been met meant that the participants had to stop and weigh themselves or think about whether they had reached the goal they had set.

*I knew that there’ll be one coming in on Sunday afternoon. And I thought, ‘I’ve just got to get that last walk in before so...I can say yes, that I’ve done the five walks* [17: 58 years]

Some women said the interaction and personalized support was important, particularly if their goal was not achieved.

I think for me, the crux of the program was, ‘what are your goals?’, ‘have you met them?’, and if you haven’t, ‘what are you going to do’….I think that was probably the most important part

24: 58 years

### Tailoring of Content and Schedules of Text Messages Was Important to Clients

The women were happy with the content, timing, and frequency of the text messages primarily because they had negotiated these with the coach and the text messages were based on their preferences.

*There was nothing that I didn’t like because it was upfront. How much SMSing do you want? What do you want? You can tell us if you don’t like it, or if it’s too much or it’s too little. So I found what we had settled on originally was fine* [3: 61 years]

And yes, because there was an in-depth conversation beforehand. We worked out together about what I would need. So I felt that I had had an input into what I wanted

9: 67 years

The participants felt the text messages were relevant to their situation and the interaction was useful as “The content of the messages changed...with whether I lost or whether I gained or nothing” (20: 62 years). “It felt very personal” (18: 62 years).

### Suggestions From Participants

A few of the women expressed a preference for continued telephone calls instead of text messaging for receiving the extended contact. They felt the text messages were not as personal as the calls and did not provide the emotional support that the telephone calls had provided. These were generally the women who would respond to goal checks with more detail than a “yes” or “no” and required researcher-generated goal check responses rather than the standard automated responses to goal check replies. Some women would have liked a mix of the text message and telephone modalities. It was often women who were not comfortable or familiar with text messaging in general who would have preferred telephone calls.

*For me personally, the telephone is good…I’m quite a communicator so I think there’s restriction with text messages and sometimes the message can be delivered quite differently to what it’s actually meant to be [23: 59 years]*

It would be nice, a mix. SMS are fine…most of the time. But there were a couple of times where I wasn’t losing weight. It would have been nice to talk to her

3: 61 years

Some preferred a different modality such as email or telephone, which allowed them to give more feedback and have more of a 2-way conversation. A couple of the participants felt that email would have been useful as an extra modality.

*Because sometimes texts are good and they’re quick, doing emails, I mean they could be a good thing as well. Because you tend to….say more [21: 44years]*

Because you can express yourself a little more than a text message. In a text message you can’t convey/focus on everything that you want

7: 65 years

## Discussion

### Principal Findings

The number of text message–delivered health interventions is exponentially increasing [18], and qualitative investigation into the user experience is an important part of their evaluation. Our exploratory study provides evidence that an extended contact intervention delivered by text messages to support the maintenance of weight loss and related behaviors is acceptable to breast cancer survivors. In particular the initial and mid-intervention tailoring calls appeared to be a key to the acceptability of the text messages. The tailoring that this allowed ensured that participants received the appropriate type, timing, and number of text messages.

The aims of an extended contact intervention are to support and sustain the behavioral changes and habits developed in the initial, intensive intervention phase [12,19]. In this study, women’s consistent reporting that the text messages acted as a prompt and kept them accountable to their goals suggested the intervention met these aims. However, without the telephone support in the initial phase of the intervention, the acceptability of the text messages might have been very different. The telephone coaching allowed rapport to be established between the participant and the coach and the development of behavioral change skills, which were a key aspect of the text messages. The text messages leveraged the rapport and skills established in the initial intervention. For some women, continued telephone contact for ongoing emotional support was a preference over the text messages. Rapport and support may be particularly important in this group of women who have been through the diagnosis and intensive treatment involved in breast cancer and all the emotional effects this has incurred.

Responding to participant text messages is time-consuming and the availability of someone to reply to these messages around the clock 7 days a week will increase the cost of such a program. Donaldson et al [10] questioned whether full automation would be impersonal. The feedback in this study suggested that receiving responses from the counsellor was important when replying to goal check text messages and that the automatic responses were generally acceptable. No negative comments arose during the interviews regarding delays for researcher-generated (nonautomated) responses when participant goal check replies were made after researcher working hours. This suggested that participants did not have an expectation for a 24-hour service.

Feedback in relation to the 6-month duration of the intervention was positive. Some women did, however, report that the text message itself rather than the content was more of a prompt toward the end. Extending the duration of text message interventions beyond 6 months may increase the likelihood of participant fatigue with the messages for some women and therefore reduce the influence and cost-effectiveness of such interventions. Developing a fully automated system, which would allow participants to self-tailor the type and frequency of text messages, might be an option for participants wanting longer-term support. Whether full automation would be as acceptable would require further investigation. In the only other study to examine preference for the length of intervention, Shaw reported a preference for the intervention to continue beyond a month [12].

Some research [20] has suggested that text messages may be more acceptable in a younger phone- and tech-savvy population. The women in this study were generally very accepting of the technology and, with the exception of a couple of participants, did not report any difficulties with the technology. Younger people may be more saturated with text messages, whereas for the women in this study, receipt of text messages may be less frequent and the unique nature may therefore act as more support. Gathering qualitative feedback from different age groups would be beneficial in planning future interventions [18].

Email was suggested by a couple of participants as an alternative support; however, this may not provide a real-time prompt to prevent certain behaviors and encourage others. Many do not access emails as frequently, and emails may be more associated with work and may not be as readily accessible if away from a computer or on holidays [21]. Some of the women preferred the support from telephone calls received in the initial intervention phase, as this enabled an easier two-way conversation. Offering alternate supports such as email and telephone calls will increase the cost of extended contact in comparison to a solely text message intervention; however, it may increase uptake and acceptability and reduce attrition [22].

### Limitations

Study participants were a group of breast cancer survivors who volunteered for the study. The high level of acceptance of the text messages may thus reflect this self-selection, with women who did not feel comfortable receiving text messages opting out of study participation. Generalizing findings to the broader adult population and, in particular, males should be done with caution. However, even outside the research context, receipt of text messages will always be an ‘opt-in’ occurrence, and thus the study results are likely generalizable to those women who would be adopters of such an intervention.

### Conclusions

Qualitative evaluation from a group of breast cancer survivors receiving extended contact via text messages suggested that this modality was effective in providing support for maintaining weight loss, physical activity, and dietary behavioral change. Importantly, this modality was seen as acceptable in this group of women. The text messages prompted healthy behaviors for participants and kept them accountable to their goals. Tailoring the number, type, and frequency of text messages was a key to the acceptance of the text messages. Providing semi-automated feedback also improved acceptability. Offering an array of support methods such as telephone support or email as an alternative to text messages may improve participation in extended contact interventions and requires further investigation.

## Appendix

Multimedia Appendix 1
