# Supplementary material for: Women’s Perceptions of Participation in an Extended Contact Text Message–Based Weight Loss Intervention: An Explorative Study
Source: JMIR Mhealth Uhealth. 2017 Feb 27;5(2):e21. doi: 10.2196/mhealth.6325 (PMC5348617; doi:10.2196/mhealth.6325)
Supplement: Multimedia Appendix 1 [file mhealth_v5i2e21_app1.pdf]

Supplementary Table 1. Feedback interview questions.

My PhD is investigating whether extended contact via text messaging to maintain lifestyle changes is feasible and acceptable to women following a weight loss program. So the aim of this part of the study is to get as much feedback as possible about using text messaging to support women.<br>Over the past six months you have received text messages from Amy/Jenny, and I want you to focus on these while I ask you some questions.<br>Please don't be afraid to share any negative experiences you had with receiving the text messages as this is the first time this type of study has been done and all comments, be they positive or negative, are extremely helpful in determining how best to move forward.<br>You are free to stop this interview at any point, and you are also free to not answer any questions which you do not feel comfortable answering.<br>*Prior text message user:*<br>Before we started this study six months ago, would you say that you were a frequent text message user?<br>Did you receive any text messages from Amy/Jenny during the initial six months of the program when you were also receiving telephone calls?<br>*Likes and dislikes*<br>Overall how did you find the experience of receiving the text messages over the last six months?<br>What did you like most about receiving the text messages? (e.g., tailored, timing and frequency self-determined) *Why?*<br>Can you recall any specific messages you found most helpful or supportive? *Why?* (e.g., goal check, ones that encouraged me to focus on my goal)<br>What did you like the least about receiving the text messages? (e.g., the frequency, too repetitive) *Why?*<br>Can you recall any specific messages you found annoying or unhelpful? *Why?* (e.g., goal check because made frustrated as didn't achieve goal)<br>*Usefulness of different types of texts*<br>Do you think the text messages helped you to stay on track to reach your goals? *If yes, How? No, why?*<br>How useful was it to receive a text asking you whether you reached your goals?<br>On average, did you respond to these?<br>When you did respond, did you like that you received a reply to this message from Amy/Jenny? Would it have mattered if you didn't?<br>If you didn't reply, what was the most common reason for not replying back to this message?<br>How useful was it to receive a text reminding you to check your weight?<br>*Tailoring of texts*<br>How did you find the number of texts you received? (e.g., too many)<br>How did you find the timing of the texts you received?<br>In regards to the number of texts and timing you received them, were they what you asked for?<br>Did you send a text asking for the frequency or timing of the way you

were receiving messaged to be changed at any point? *Did making that change improve the helpfulness of the texts? If you didn't send a text asking to change this but you wanted to, why didn't you?* (e.g., too difficult, too hard to explain etc.)
How did you find the language used in the text messages?
Was the abbreviation of words ok? You (u); Be (b); To (2); Your (ur); See (c)
Did you feel the content of the messages were sufficiently personalised to you? How?
To what extent did the content of the messages you received over the last six months feel relevant to your situation?
Did the consistency with which you read text messages change over the last six months? (e.g., read more at beginning and less at the end)
On average over the last six months, what percentage of the time would you say you *didn't* read a text message?
12-week check in call
At any point over the last six months did you wish you could just talk to Amy/Jenny?
To what extent did you find the telephone call half way through the last 6 months useful?
Would you have been happy to continue contact via text only or do you think it was important to be able to talk to Amy or Jenny at that point?
Future suggestions
Do you have any suggestions about how to improve the text messages so that they would be more helpful to women following the first part of the LWaBC program?
Do you have any other feedback or comments about what you liked or disliked, or any comments about your experiences with receiving the text messages over the last six months?
